# Design and Optimization of Piezoelectric Cantilever Beam Vibration Energy Harvester

**DOI:** 10.3390/mi13050675

**Published:** 2022-04-26

**Authors:** Qiuyu Xu, Anran Gao, Yigui Li, Yan Jin

**Affiliations:** 1 College of Sciences, Shanghai Institute of Technology, Shanghai 201418, China; xqy130@hotmail.com (Q.X.); ygli@sit.edu.cn (Y.L.); 2 State Key Laboratory of Transducer Technology, Shanghai Institute of Microsystem and Information Technology, Chinese Academy of Sciences, Shanghai 200050, China; 3 Department of Electronics, East China Normal University, Shanghai 200241, China

**Keywords:** optimization, FEA, PEH, hollow structure, high energy density

## Abstract

Piezoelectric cantilever beams are commonly utilized to harvest energy from environmental vibrations due to their simple structures. This paper optimizes a single crystal trapezoidal hollow structure piezoelectric cantilever beam vibration energy harvester with a copper substrate to achieve high energy density at a low frequency. Finite element analysis (FEA) is adopted to optimize the size of the copper substrate at first, and the piezoelectric energy harvester (PEH) is further optimized with a trapezoidal hollow structure under the optimal size of the copper substrate. The developed PEH with a trapezoidal hollow structure (L_a_ = 20 mm, L_b_ = 15 mm, and L_h_ = 40 mm), with a copper substrate of 80 mm × 33 mm × 0.2 mm, can obtain the best output performance. Under the condition of 1 g acceleration, the resonance frequency and peak voltage output were 23.29 Hz and 40.4 V, respectively. Compared with the unhollowed PEH, the developed trapezoidal hollow structure PEH can reduce its resonant frequency by 12.18% and increase output voltage by 34.67%, while also supplying a power density of 7.24 mW/cm^3^. This study verified the feasibility of the optimized design through simulation and experimental comparison.

## 1. Introduction

Despite the fact that many electronic devices still rely solely on chemical batteries for power in the face of the miniaturization trend of embedded electronic devices and sensors [1,2,3,4,5,6,7,8], traditional batteries, due to their massive size [5,6,9] and their need for timely replacement or recharging [6,7,8,10,11,12], no longer meet the power needs of some electronic devices [8,13,14,15]. With the development of electronic integration technology and microelectromechanical technology, the power consumption of some commonly used sensing and transmitting modules can be reduced to hundreds of nW, which makes it possible to use energy harvesting technology to power low power electronic devices [16]. Harvesting energy from the environment to power electronic devices or charge batteries has the potential to improve this situation. As reported, solar, wind, geothermal, water, and vibration energies can all be harvested from our environment [8,17,18,19,20]. Vibrations can be found all around us, such as the vibration of a car passing by, a machine in operation, or a tree branch in the wind. Vibration energy harvesting can, thus, be applied in a variety of environments [7,8,13,21,22].

Diverse transduction mechanisms based on piezoelectric, electromagnetic, and electrostatic generation principles have been utilized to convert vibration into electrical energy [13,14,23,24,25,26,27,28,29]. Due to their higher energy density and simpler structure [4,19], piezoelectric energy harvesters (PEHs) have attracted attention [5,6,7,10,12,15]. In addition, piezoelectric materials are well suited for flexible and stretchable devices, and can be easily scaled in micro and nano devices [4,8,12,30].

When vibrations from the external environment drive the deformation of the piezoelectric material, opposite charges are generated on two opposite surfaces of the piezoelectric material due to the piezoelectric effect [31]. A voltage difference is created between the two opposing surfaces, causing the charges in the circuit to travel in a specific direction. A current can be detected in the circuit. Piezoelectric energy harvesters with cantilever beam structures are widely applied because of their simple structure and significant deformation under vibration [5,12].

In order to improve the output performance of the piezoelectric cantilever, researchers have made many improvements to the cantilever. Karadag, C.V. et al. [32] optimizes the shape of the cantilever beam and adopts a curved beam. In the two cases of the beam with no tip mass and a 5 g tip mass, compared with the regular triangular beam, the optimized beam shows a 22% and 29% increase in strain uniformity, respectively. Wu, H. et al. [33] designed a two-degrees-of-freedom piezoelectric cantilever beam, comprising of one main cantilever beam and an inner secondary cantilever beam. By adjusting the masses, the first two resonant frequencies can be tuned very close, resulting in a wider effective operating bandwidth. Xiong, Y. et al. [34] designed a cantilever beam with a rectangular hollow middle layer, which reduced the resonance frequency of the cantilever beam and increased the output voltage. Wang, L. et al. [35] designed a hollow triangular piezoelectric cantilever beam suitable for the random low frequency vibration environment of transportation infrastructure. Wang, B. et al. [36] designed a piezoelectric cantilever beam with a trapezoidal hole, and this piezoelectric cantilever beam obtained a power density of 8.932 mW/cm^3^ at an acceleration of 1.5 g. The triangular hole, rectangular hole, and trapezoidal hole were compared in Wang B’s research, and the PEH with a trapezoidal hole was proved to have the best performance. However, for PEH with a trapezoidal hole, the influence of the direction and area of the trapezoid on the output have yet to be investigated in detail.

However, piezoelectric energy harvesting technology still faces some challenges. Harvesters with low frequency, high power density, and good stability are urgently needed to meet the requirements of energy development [15,37]. In recent years, the main contradiction is that the vibration source requires the device to match a lower resonance frequency. In addition, material optimization, substrate replacement, and geometry change are the main methods currently utilized for the optimization of harvesters [38,39]. However, to date, there have been limited systematic studies on cantilever beam structure-based PEHs.

In this paper, based on the establishment of the rectangular piezoelectric cantilever model, the effect of size of the copper substrate on the output of the piezoelectric cantilever beam is initially analyzed via finite element analysis (FEA), from which the optimal size of the substrate is determined. The trapezoidal hollow design is then carried out on the cantilever beam with the optimal copper substrate, and the position and size of the trapezoidal holes are optimized to achieve the maximum voltage output. The influence of the hollow structures on the stress distribution of harvesters is analyzed in the resonance state. The designed hollow harvesters with optimized copper substrate realize the improvement and expansion of the stress distribution of the piezoelectric layer. The fabricated trapezoidal hollow structure PEH obtains the best power density at a lower resonance frequency, which makes its application more flexible.

## 2. Design and Simulation

**Figure 1:**
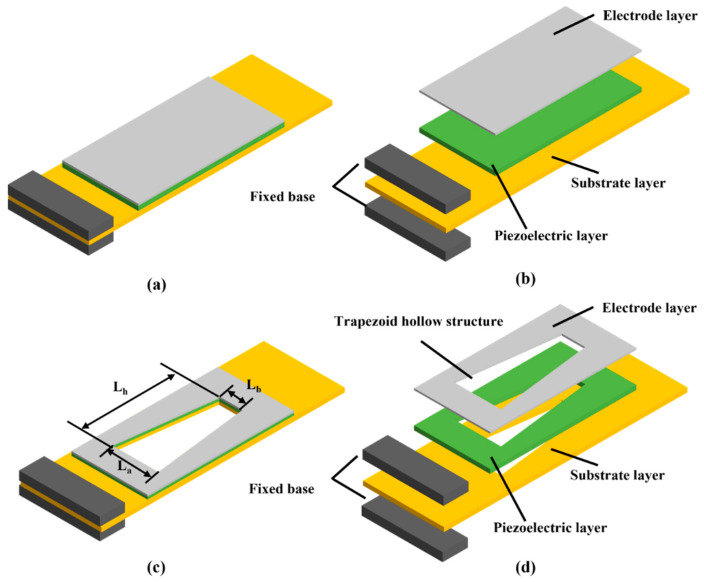
Schematic diagram of PEHs: (**a**) the traditional structure of PEH; (**b**) components of traditional PEH; (**c**) the proposed trapezoidal hollow structure of PEH with a copper substrate; (**d**) components of proposed PEH with a copper substrate.

The structure and components of a traditional PEH is shown in Figure 1a,b, respectively. As can be seen, the left end is the fixed end of the cantilever, which is fastened to the excitation platform by fixed bases, and the right end is the free end, which would shake up and down with the vibration. The cantilever beam can be deformed by force due to external vibration, so as to achieve different vibration states and produce matching outputs. An electrode layer, a piezoelectric layer, and a substrate layer are the three primary layers of the PEH. In this work, a silver layer, a PZT layer, and a copper substrate are adopted as the electrode layer, the piezoelectric layer, and the substrate layer, respectively, and size of copper substrate is optimized at first. Figure 1c,d represent a high-performance PEH with an optimized copper substrate and a trapezoidal hollow structure, wherein L_a_, L_b_, and L_h_ in Figure 1c represent the length of the bottom near the fixed end of the PEH, the length of the other bottom near the free end of the PEH, and the height of the trapezoid, respectively.

The material types and parameters of PEHs in the simulation and experiment are shown in Table 1. The piezoelectric coefficient matrix, elastic coefficient matrix, and dielectric coefficient matrix of the PZT-5H are shown in Equations (1)–(3), respectively.

(1) $$\varepsilon_{r}=\{\begin{array}{ccc} 3130 & 0 & 0\\ 0 & 3130 & 0\\ 0 & 0 & 3400 \end{array}$$

(2) $$d=\{\begin{array}{cccccc} 0 & 0 & 0 & 0 & 6.6 & 0\\ 0 & 0 & 0 & 6.6 & 0 & 0\\ -1.86 & -1.86 & 6.7 & 0 & 0 & 0 \end{array}\times 10^{-10}\;C/N$$

(3) $$s=\{\begin{array}{cccccc} 16.5 & -4.78 & -8.45 & 0 & 0 & 0\\ -4.78 & 16.5 & -8.45 & 0 & 0 & 0\\ -8.45 & -8.45 & 20.7 & 0 & 0 & 0\\ 0 & 0 & 0 & 43.5 & 0 & 0\\ 0 & 0 & 0 & 0 & 43.5 & 0\\ 0 & 0 & 0 & 0 & 0 & 42.6 \end{array}\times 10^{-12}\;m^{2}/N$$

The body loads in the solid mechanics module are simulated for external vibrations, and the entire model is meshed to divide it into many small, regularly shaped elements. The derivation process for the acceleration formula of body load in the simulation is as follows.

The force (*F*) applied to each element has the relationship with mass (*m*) and acceleration (*a*) shown in Equation (4), as follows:

(4) F=ma

Meanwhile,

(5) m=ρV

where *ρ* is the density of each cell and *V* is the volume of the corresponding cell.

(6) a=acc·gsin(2πft)

where *acc* is the coefficient of acceleration, *g* is the acceleration of gravity (1 *g* ≈ 9.795 m/s^2^), *f* is the frequency of excitation vibration and *t* is the time of vibration.

(7) F=acc·ρVg·sin(2πft)

Therefore, the force received by the model per unit volume is

(8) $$F_{V}=acc\cdot \rho g\cdot \sin\left(2\pi ft\right)$$

In the simulation, acceleration of vibration excitation can be changed directly by adjusting the value of *acc*.

**Figure 2:**
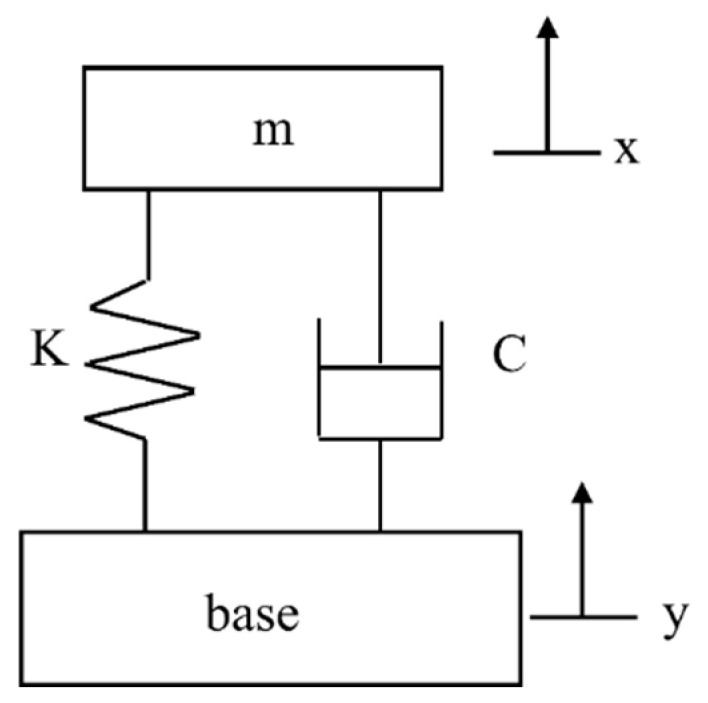
Diagram of a spring-mass-damper base excitation system.

The vibration mass-spring-damper base excitation system has been used to describe the energy that can be obtained from the vibration source [40]. The model is shown in Figure 2. The model assumes that the mass of the vibration source is much larger than the seismic mass in the generator, and the vibration source is an infinite power source [41].

The displacement of base is given as follows:

(9) $$y\left(t\right)=Y_{0}sin\omega t$$

The relative displacement of mass is given as follows:

(10) z(t)=x(t)−y(t)

where, *x* is the displacement of seismic mass.

The equation of motion for the vibrating system is given as follows:

(11) $$m\ddot{z}\left(t\right)+c\dot{z}\left(t\right)+kz\left(t\right)=-m\ddot{y}\left(t\right)$$

where, *m* is the seismic mass, and c and k are damping constant and spring constant, respectively.

The total power dissipated in damper under sinusoidal excitation is given as follows:

(12) $$P_{T}\left(\omega \right)=\frac{m\xi Y^{2}{\left(\frac{\omega }{\omega_{n}}\right)}^{3}\omega^{3}}{{\left[1-{\left(\frac{\omega }{\omega_{n}}\right)}^{2}\right]}^{2}+{\left[2\xi \left(\frac{\omega }{\omega_{n}}\right)\right]}^{2}}$$

where $\xi =c/2\sqrt{mk}$ is the total damping ratio of the system, and $\omega^{2}=k/m$ is the system resonant frequency.

When the system operates at resonance frequency, the maximum power is given as follows:

(13) $$P_{max}=\frac{mY^{2}\omega^{3}}{4\xi }$$

It can be seen from Equation (13) that the power of piezoelectric cantilever can be improved by reducing the damping of the cantilever, increasing the mass, and increasing the excitation amplitude.

**Figure 3:**
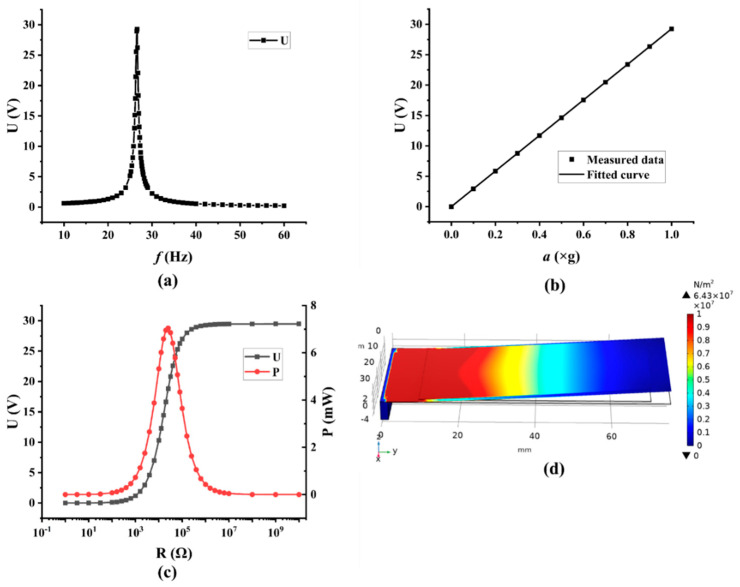
Simulation results: (**a**) voltage output at different frequencies; (**b**) voltage output at different accelerations; (**c**) voltage output and power output with different load resistances; (**d**) surface stress distribution.

A piezoelectric cantilever beam model with the dimensions shown in Table 2 was finally built, and the electrode layer was neglected in the finite element analysis (FEA). When the acceleration was equal to 1 *g* (1 *g* ≈ 9.795 m/s^2^), the simulation results of the model are shown in Figure 3.

Through frequency sweeping, the voltage output of the piezoelectric cantilever beam at different frequencies is calculated, and the results are depicted in Figure 3a, wherein the frequency range is from 10 Hz to 60 Hz. As can be seen, the resonance frequency and the peak output voltage are 26.6 Hz and 29.272 V, respectively. Subsequently, under the condition of the resonance frequency, the peak voltage output at diverse accelerations is simulated, wherein the acceleration ranges from 0 *g* to 1.0 *g* with a step size of 0.1 *g*, and the result is shown in Figure 3b. A linear relationship between the peak voltage output (U) and the acceleration (a) is obtained, with the regression equation of U = 29.27229 a. The relationship between the output voltage and the external load resistance is tested, and then the relationship between the output power and the external load resistance is calculated correspondingly, and the results are shown in Figure 3c. As can be seen, as the value of the external load resistance increases, the voltage output of the PEH at the resonance frequency would increase accordingly, at first, and then would tend to stabilize, while the power output would increase at first and achieve a peak, and then decrease. According to the maximum power transfer theorem [42], when the value of external load resistance is equal to the resistance of the piezoelectric cantilever beam, the peak output power would be achieved and, therefore, the resistance of the piezoelectric cantilever beam can be calculated to be 24.2 kΩ. The stress distribution of the piezoelectric layer at resonance frequency on the outside of the surface of the piezoelectric layer is simulated, and the result is presented in Figure 3d. It is found that the stress level is concentrated near the fixed end and decreases toward the free end of the cantilever.

## 3. Optimization

The output of the PEH can be improved by optimizing both the geometry and materials of the piezoelectric cantilever, which would have an impact on its force situation. In order to improve the output of the PEH, two steps are carried out in this work, that is, the size of the copper substrate is optimized firstly, and then a trapezoidal hollow structure is proposed to further improve the output.

**Figure 4:**
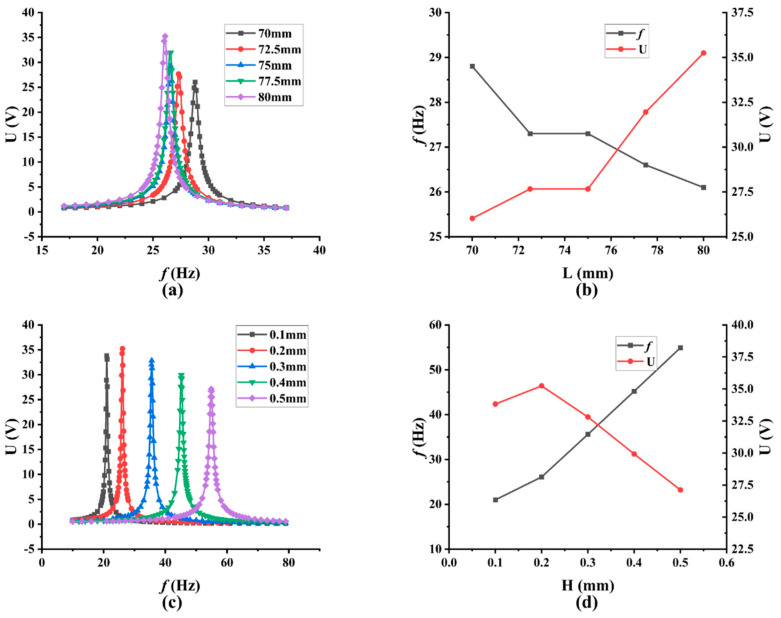
Simulation results at diverse copper substrate size: (**a**) voltage output at different frequencies with diverse lengths; (**b**) resonance frequency and voltage output with different lengths; (**c**) voltage output at different frequencies with diverse thicknesses; (**d**) resonance frequency and voltage output with different thicknesses.

Copper substrates with a 33 mm width and a 0.2 mm thickness were selected, and the size of the length was selected in the range of 70 mm to 80 mm, with a step size of 2.5 mm. Finite element analysis was then carried out to obtain their resonance frequencies and peak voltage outputs, and the results are summarized in Figure 4a,b. As can be seen from the results, as size of the length increased from 70 mm to 80 mm, the corresponding resonance frequency would decrease from 28.8 Hz to 26.1 Hz, while the peak voltage output would increase from 26.026 V to 35.243 V. When the length of the cantilever beam gradually increases, the resonant frequency decreases, but in the actual design, the length of the cantilever beam is too large, which will lead to large bending or even breakage due to the gravity of the structure itself. To achieve the maximum voltage output, 80 mm is determined as the length of the copper substrate. Based on the selected copper substrate with a 33 mm width and a 80 mm length, the PEHs with copper substrates with five different thicknesses of 0.1 mm, 0.2 mm, 0.3 mm, 0.4 mm, and 0.5 mm were simulated, and the results are presented in Figure 4c,d. As the thickness of the copper substrate increased, the resonance frequency of the PEH was found to increase continuously, while the peak voltage output would increase, at first, to a maximum value of 35.243 V with the thickness of 0.2 mm, and then decrease as the thickness increased further. As a result, the final thickness of copper substrate is determined to be 0.2 mm. Therefore, the final optimized size of the copper substrate of the piezoelectric cantilever beam is determined to be 80 mm × 33 mm × 0.2 mm. The above performance comparison is summarized in Table 3.

**Figure 5:**
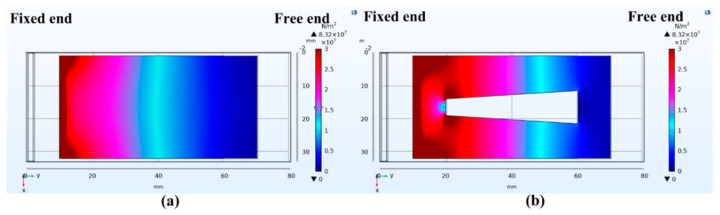
Surface stress distribution of piezoelectric cantilever beam: (**a**) unhollowed structure; (**b**) with a trapezoidal hollow structure.

The voltage output of a piezoelectric cantilever is further increased by the hollowing procedure. Figure 5a,b show the surface stress distribution of the piezoelectric layer on both PEHs without and with a trapezoidal hollow, respectively, under the same color scale. It is found that, the redder of the color, the higher of the stress density would be obtained, and the bluer of the color, the lower of the stress density would be obtained. From the surface stress distribution presented in Figure 5a,b, it can be observed that the region of the unhollowed cantilever beam with the high stress density (partial red region) is main concentrated near the fixed end, and the region of the hollowed cantilever beam with high stress density expands toward the free end. As the high stress area is enlarged by the hollowed design, the output voltage of the PEH would increase accordingly.

**Figure 6:**
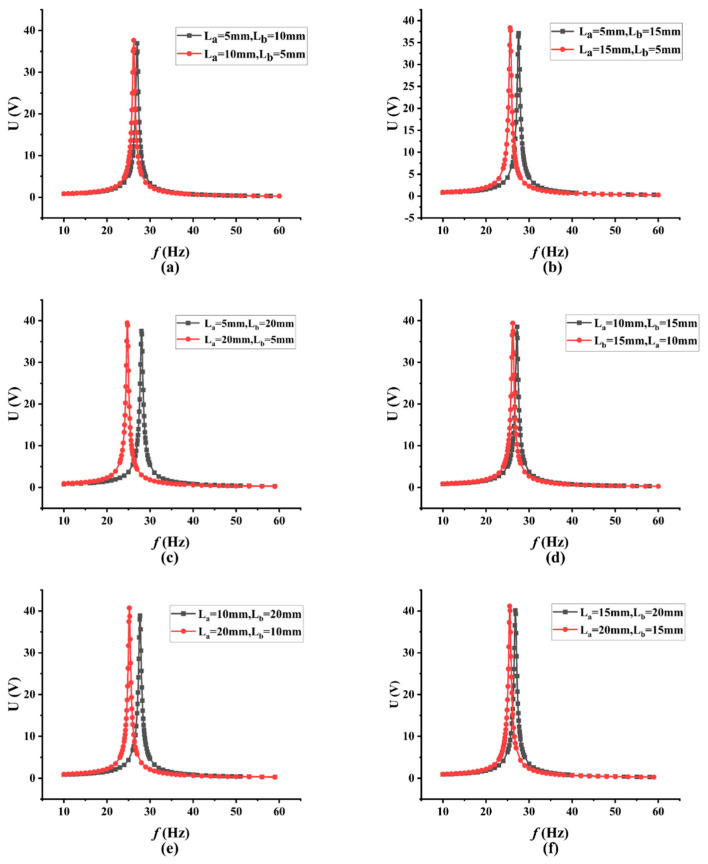
Comparison of output voltages of piezoelectric cantilever beams with different trapezoidal holes.

The hollow design of isosceles trapezoid is then replicated on the PEH. The size of the trapezoid hollow includes the length of the bottom near the fixed end (L_a_), the length of the other bottom near the free end (L_b_), and the height of the trapezoid (L_h_), which has been shown in Figure 1c. As the trapezoid hollow structure with too large an area may cause the fracturing of the piezoelectric layer in practical application, the height of the trapezoid hollow is determined to be 40 mm, that is L_h_ = 40 mm. The lengths of the two bottoms L_a_ (near the fixed end) and L_b_ (near the free end) are selected in the range of 5 mm to 20 mm, with a step size of 5 mm. Twelve PEH models with trapezoidal hollow holes of diverse sizes were finally obtained, and were divided into six groups with two models in one group. The two models in each group have the opposite bottom size. For example, when one model has the size of L_a_ = 5 mm and L_b_ = 10 mm (L_a_ < L_b_), the other one must have the size of L_a_ = 10 mm and L_b_ = 5 mm (L_a_ > L_b_), respectively. The results of the six groups of PEH models are summarized in Table 4 and Figure 6. In Figure 6, the black curve represents the condition of L_a_ < L_b_, and the red curve represents the condition of L_a_ > L_b_. It can be concluded from Figure 6 that the peak voltage output would be larger, and the resonance frequency would be smaller, under the condition of L_a_ > L_b_. Therefore, L_a_ should be designed to be larger than L_b_ in order to obtain the optimized peak voltage output and the lower resonance frequency.

**Figure 7:**
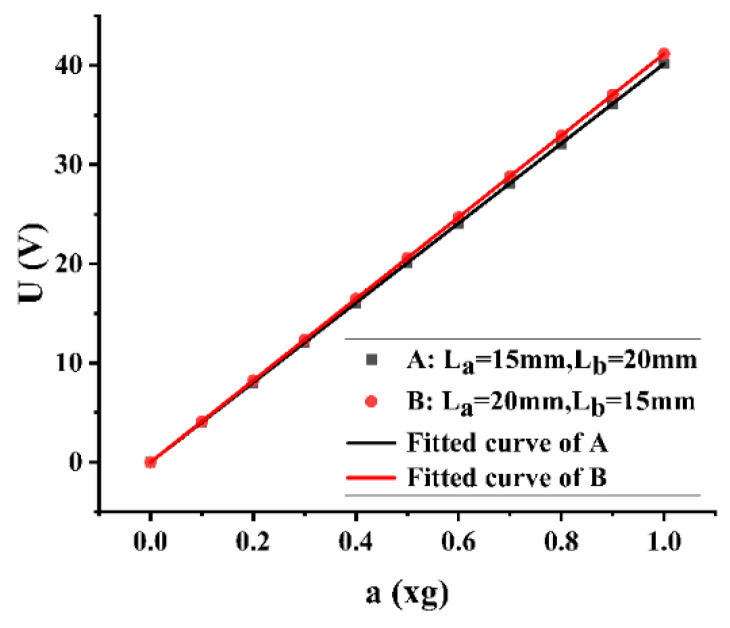
The voltage output of the six group at different accelerations.

As shown in Figure 7, both A (L_a_ = 15 mm, L_b_ = 20 mm) and B (L_a_ = 20 mm, L_b_ = 15 mm) are cantilever beams with trapezoidal holes. Their output voltage (U) has a linear relationship with acceleration (a), and the corresponding equations are U = 40.17504 a and U = 41.18206 a, respectively.

## 4. Experiment

**Figure 8:**
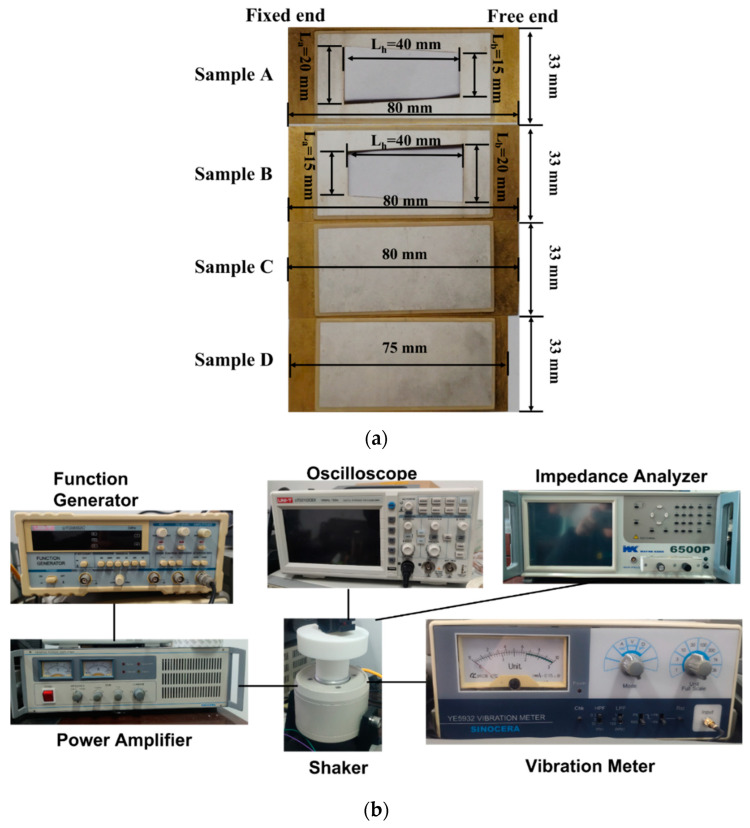
(**a**) Experimental samples: A, with a trapezoidal hollow hole (L_a_ = 20 mm, L_b_ = 15 mm); B, with a trapezoidal hollow hole (L_a_ = 15 mm, L_b_ = 20 mm); C, with an optimized copper substrate size (80 mm × 33 mm × 0.2 mm); D, with an unoptimized copper substrate size l (75 mm × 33 mm × 0.2 mm). (**b**) Experimental platforms.

According to the results summarized in Table 4, the two models in the sixth group achieve the highest peak voltage output, with values of 40.175 V and 41.182 V, respectively. The PEHs with the above two trapezoidal hollow structure are fabricated, shown as Sample A and Sample B in Figure 8a. The unhollowed samples with an optimized copper substrate with a size of 80 mm × 33 mm × 0.2 mm and an unoptimized copper substrate with a size of 75 mm × 33 mm × 0.2 mm were also fabricated as a comparison, shown as Sample C and Sample D in Figure 8a.

The preparation process of the samples is as follows: firstly, the PZT and copper were cleaned in an ultrasonic cleaner for 10 min and put into an 80 °C oven to dry water; then the surfaces of the PZT and copper were polished, a layer of conductive silver glue was brushed on the copper, and the PZT was placed on the copper substrate; the sample was placed in a vacuum oven at 200 °C for 3 h (a heavy object was always pressed on the sample to make the sample under a uniform pressure); finally, the sample was gradually cooled down. During this process, the temperature was decreased by 30 °C each time, and the temperature was maintained for half an hour until the temperature reached room temperature. A laser (RZY-BX-10B) was used to add a trapezoidal hollow structure on the piezoelectric cantilever.

These four samples were tested by the experimental platform as illustrated in Figure 8b, which consists of a dual-channel oscilloscope (UNI-T UTD2102CEX), a function generator (UNI-T UTG9002C), a vibration meter (SINOCERA YE5932), a power amplifier (YE5872A), an impedance analyzer (WK6500P), and a vibrator (JZK-5).

**Figure 9:**
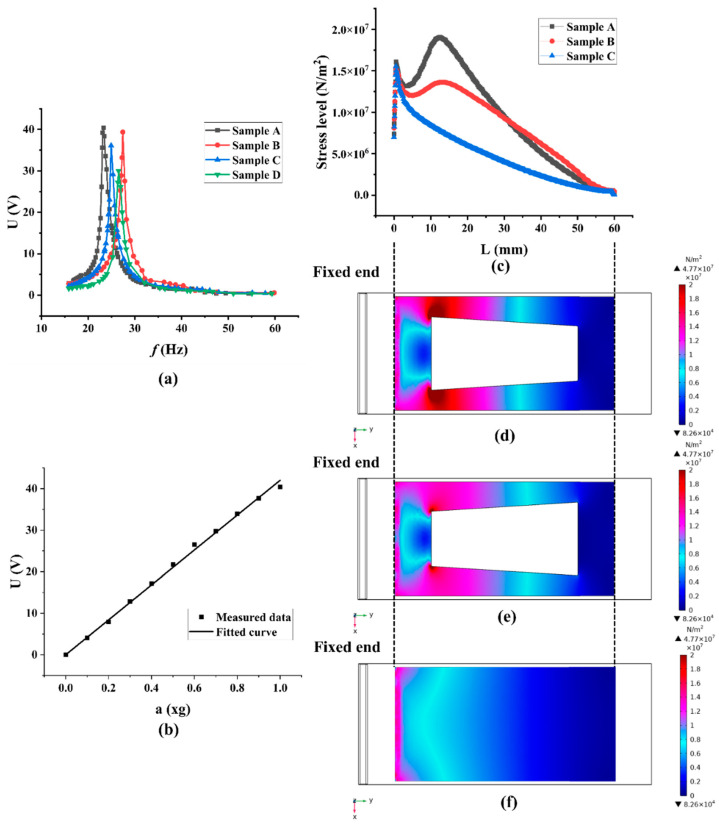
(**a**) Comparison of experimental results; (**b**) Voltage output of Sample A at different accelerations; (**c**) Stress level on the piezoelectric material from the fixed end to the free end; (**d**) Stress level distribution of Sample A; (**e**) Stress level distribution of Sample B; (**f**) Stress level distribution of Sample C.

The test results for the above four samples are shown in Figure 9a and Table 5. Under 1 *g* (*g* = 9.795 m/s^2^) acceleration excitation conditions, the resonance frequency and peak voltage output of Sample A (with the trapezoidal hollow structure (L_a_ = 20 mm, L_b_ = 15 mm, L_h_ = 40 mm), with a copper substrate size of 80 mm × 33 mm × 0.2 mm), Sample B (with the trapezoidal hollow structure (L_a_ = 15 mm, L_b_ = 20 mm, L_h_ = 40 mm), with a copper substrate size of 80 mm × 33 mm × 0.2 mm), Sample C (an unhollowed structure, with a copper substrate size of 80 mm × 33 mm × 0.2 mm) and Sample D (an unhollowed structure, with a copper substrate size of 75 mm × 33 mm × 0.2 mm) can be obtained as 23.29 Hz, 27.42 Hz, 24.92 Hz, 26.52 Hz and 40.40 V, 39.37 V, 36.12 V, and 30.00 V, respectively. As can be calculated, the peak voltage output is increased by 20.4%, and resonant frequency is reduced by 6.03% after the copper substrate size is optimized (comparison between Sample C and Sample D). Moreover, it is found that the trapezoidal hollow design of the PEH effectively improves the voltage output of the PEH, as the peak voltage outputs of both Sample A and Sample B are higher than that of Sample C. In the case of the trapezoidal hollow design, the peak voltage output of sample A is increased by 2.62%, 11.85%, and 34.67% compared with sample B, sample C, and sample D, respectively, and resonant frequency was reduced by 15.06%, 6.54%, and 12.18%, respectively. Therefore, the long bottom of the trapezoidal hole should be placed close to the fixed end of the PEH, that is L_a_ > L_b_.

According to Wang B’s research [36], the output voltage of PEH can be improved by adding a trapezoidal hollow structure. The peak output voltage of Sample B (Wang B’s method) is increased by 9.00% compared with the unhollowed PEH (Sample C). On this basis, by adjusting the direction of the trapezoidal hole, the output voltage of Sample A is 11.85% higher than that of the unhollowed PEH (Sample C).

The output voltage of piezoelectric cantilever beam (Sample A) with a trapezoidal hole under different accelerations is shown in Figure 9b. The measured data are fitted to obtain the corresponding equation of U = 42.03117 a.

The three samples, A, B, and C, all have the same size of copper substrates of 80 mm × 33 mm × 0.2 mm. The variation of the stress level on their piezoelectric layers in the direction from the fixed end to the free end of the PEH is calculated and shown in Figure 9c, while the stress distributions of piezoelectric layers on the three samples are presented in Figure 9d, Figure 9e and Figure 9f, respectively. As can be concluded from Figure 9c, from the fixed end to the free end, the stress level in the piezoelectric layer of the unhollowed PEH (Sample C) increased rapidly at first, quickly reached a maximum value, and then decreased gradually. Unlike the unhollowed PEH (Sample C), the stress level on the PEHs with a trapezoidal hollow structure (Sample A and Sample B) increased to another maximum value near the trapezoidal bottom near the fixed end, forming a second peak stress level before finally gradually decreasing. It can be seen from Figure 9c that the second stress level peak is higher for Sample A than for Sample B. It can also be seen in Figure 9d,e that, at around L = 10 mm, Sample A is redder in color and receives a higher stress level than Sample B. Therefore, Sample A can achieve a higher peak voltage output.

The power output can be calculated by the equation presented as $P=U^{2}/\left(8\cdot R\right)$ [36]. Matched loading resistance of Samples A, B, C, and D were measured to be 45.42 kΩ 46.13 kΩ, 27.1 kΩ, and 27.5 kΩ, respectively, and their corresponding maximum power densities were calculated to be 7.24 mW/cm^3^, 6.77 mW/cm^3^, 6.69 mW/cm^3^, and 4.72 mW/cm^3^, respectively.

**Figure 10:**
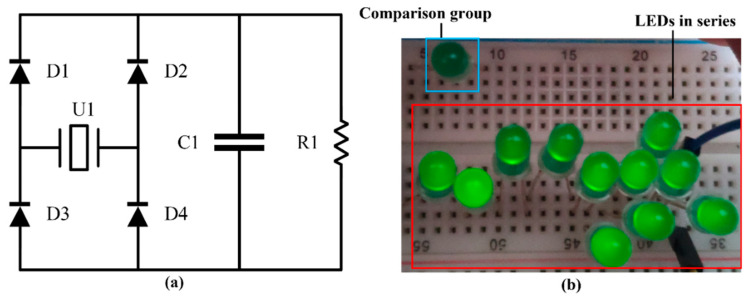
Simple application: (**a**) Circuit; (**b**) the series LEDs are lit.

Figure 10 shows a simple application of a piezoelectric cantilever beam. Figure 10a shows a circuit that converts alternating current into direct current, in which U1 is a piezoelectric cantilever beam, D1, D2, D3, and D4 are four diodes, C1 is a filter capacitor, and R1 is a load. In Figure 10b, the LED in the blue box is not connected to the circuit as a comparison group, and the series LEDs in the red box are lit by piezoelectric cantilevers. Finally, the maximum output power of Sample A is 4.49 mW, which can satisfy some ultra-low power consumption electronic devices. The energy consumption of some low power device is shown in Table 6 [16].

The vibration frequency of many environmental vibration sources in nature is very low. The characteristic frequency of the piezoelectric cantilever optimized in this paper is more suitable for the car engine (above 20 Hz) [43], and is also close to the vibration frequency of indoor wooden stairs caused by foot traffic (about 27 Hz) [44].

## 5. Conclusions

In summary, the structure and characteristics of a piezoelectric cantilever beam energy harvester are investigated and optimized in this study. The piezoelectric cantilever beam is optimized by adjusting the size of the copper substrate to provide a higher output of the PEH without initially changing the piezoelectric material, and a copper sheet of 80 mm × 33 mm × 0.2 mm is finally selected as the optimal size. Through finite element simulation and experimental measurements, the size of a trapezoidal hollow PEH with the optimized copper substrate is investigated in detail. When the length of bottom of the trapezoidal hole near the fixed end of the PEH is larger than near the free end (L_a_ > L_b_), the output voltage is higher. As a result, a PEH with a trapezoidal hollow structure with a size of L_a_ = 20 mm, L_b_ = 15 mm and L_h_ = 40 mm is obtained. Compared with the unhollowed PEH, under the condition of 1 g acceleration, the developed PEH with a trapezoidal hollow structure hole can reduce its resonant frequency by 12.18% and increase its output voltage by 34.67%, while the PEH can supply a power density of 7.24 mW/cm^3^.

## Figures and Tables

**Table 1:** Basic parameters of the material.

Structure	Electrode Layer	Piezoelectric Layer	Substrate Layer
Material	Ag	PZT-5H	copper
Density/(kg/m^3^)	10,500	7500	8780
Yang’s modulus/(10^10^ N/m^2^)	83	5.6	11.2
Poisson’s ratio	0.37	0.36	0.35

**Table 2:** Dimension parameters.

Title 1	Piezoelectric Layer	Substrate Layer
Length/(mm)	60	75
Width/(mm)	31	33
Thickness/(mm)	0.2	0.2

**Table 3:** Performance comparison of cantilever beams with various sizes of copper substrates.

Copper Substrate Size		Output	
Length (mm)	Thickness (mm)	Resonance Frequency (Hz)	Peak Output Voltage (V)
70	0.2	28.8	26.026
72.5		27.3	27.664
75		26.6	29.272
77.5		26.6	31.954
80		26.1	35.243
80	0.1	21	33.826
	0.2	26.1	35.243
	0.3	35.6	32.816
	0.4	45.2	29.927
	0.5	54.9	27.121

**Table 4:** Results of cantilever beams with diverse sizes of trapezoidal hollow holes.

Group	L_a_ (mm)	L_b_ (mm)	Resonance Frequency (Hz)	Peak Output Voltage (V)
1	5	10	27.0	36.937
	10	5	26.2	37.646
2	5	15	27.6	37.147
	15	5	25.6	38.385
3	5	20	28.0	37.543
	20	5	24.7	39.499
4	10	15	27.2	38.562
	15	10	26.2	39.441
5	10	20	27.7	38.91
	20	10	25.2	40.748
6	15	20	26.8	40.175
	20	15	25.5	41.182

**Table 5:** Test results before and after optimization.

Sample Number	Copper Substrate Size		Trapezoidal Hollow Hole		Resonance Frequency (Hz)	Peak Voltage Output (V)
	Length (mm)	Thickness (mm)	L_a_ (mm)	L_b_ (mm)		
A	80	0.2	20	15	23.29	40.40
B	80	0.2	15	20	27.42	39.37
C	80	0.2	unhollowed structure		24.92	36.12
D	75	0.2	unhollowed structure		26.52	30.00

**Table 6:** Energy consumption of some low power devices [16].

Devices	Clock & Timer	Temperature & Humidity Sensors	Gas & Air Quality Sensors
Energy consumption (μW)	0.1–1	1–10	10–100

## Data Availability

Data available on request due to restrictions, e.g., privacy or ethical. The data and material presented in this study are available on request from the corresponding author.
